# Neuroprotective effect of silymarin-loaded nanoliposomes against monosodium glutamate-induced cerebellar motor deficit and Purkinje cell damage in experimental rats via PI3K/AKT pathway activation

**DOI:** 10.3389/fmolb.2025.1621240

**Published:** 2025-07-14

**Authors:** Medhat Taha, Ahmed Alzahrani, Omer Abdelbagi, Rehab M. Bagadood, Naeem F. Qusty, Rami Obaid, Mohammad El-Nablaway, Tourki A. S. Baokbah

**Affiliations:** ^1^ Department of Anatomy, Al- Qunfudah Medical College, Umm Al-Qura University, Al-Qunfudhah, Saudi Arabia; ^2^ Department of Anatomy and Embryology, Faculty of Medicine, Mansoura University, Mansoura, Egypt; ^3^ Department of Biochemistry, Al-Qunfudah Medical College, Umm Al-Qura University, Makkah, Saudi Arabia; ^4^ Department of Pathology, Qunfudah Faculty of Medicine, Umm-Al-Qura University, Makkah, Saudi Arabia; ^5^ Department of Clinical Laboratory Sciences, Faculty of Applied Medical Sciences, Umm Al-Qura University, Makkah, Saudi Arabia; ^6^ Department of Medical Genetics, Faculty of Medicine at Al-Qunfudah, Umm Al-Qura University, Al-Qunfudhah, Saudi Arabia; ^7^ Department of Basic Medical Sciences, College of Medicine, AlMaarefa University, Riyadh, Saudi Arabia; ^8^ Department of Medical Biochemistry, Faculty of Medicine, Mansoura University, Mansoura, Egypt; ^9^ Department of Medical Emergency Services, College of Health Sciences-AlQunfudah, Umm Al-Qura University, Al-Qunfudhah, Saudi Arabia

**Keywords:** cerebellar toxicity, monosodium glutamate, silymarin nanoliposomes, oxidative stress, neuroinflammation, apoptosis

## Abstract

**Background and aim:**

This study investigated the ameliorative effect of Silymarin-nanoliposome (SLNPs) against monosodium glutamate (MSG)-induced cerebellar toxicity, illuminating its impact on motor coordination.

**Methods:**

Forty male Wistar albino rats were divided into four groups. Group I (control group): rats received 2 mL of 0.9% NaCl solution; Group II (SLNPs group): rats received SLNPs with a dose of 500 μg/kg bw orally; Group III (MSG group): rats received 3.5 mg/kg bw of MSG intraperitoneally; and Group IV: rats received combined treatment of MSG + SLNPs for ten consecutive days.

**Results:**

MSG-induced cerebellar motor incoordination is represented by increased falls in rats and decreased tow latency spent on the rotarod test. Moreover, MSG altered cerebellar histological structure and significantly (p < 0.05) decreased antioxidant system activity and protein levels of phosphorylated phosphoinositide 3-kinases (p-PI3K), phosphorylated protein kinase B (p-AKT), and brain-derived neurotrophic factor (BDNF). Additionally, there is a decrease in the immunoexpression of nuclear factor erythroid 2-related factor 2 (Nrf2) and gene expression of heme oxygenase-1 (HO-1), tropomyosin receptor kinase B (TrkB), and anti-apoptotic B-cell lymphoma-2 (Bcl-2), alongside an increase in the sera and protein levels of proinflammatory cytokines tumor necrosis factor-alpha (TNF-α), interleukin-6 (IL-6), interleukin-1 beta (IL-1β), immunoexpression of glial fibrillary acidic protein (GFAP), nuclear factor kabba beta (NF-κB), caspase-3, and gene expression of proapoptotic Bax. However, SLNPs prevented MSG-induced cerebellar toxicity, improving motor coordination and morphological structure by enhancing antioxidant, anti-inflammatory, and anti-apoptotic activity by stimulating the PI3K/AKT pathway.

**Conclusion:**

The current study indicated that SLNP administration protects against MSG-induced cerebellar damage, preventing cerebellar oxidative stress, inflammation, and apoptosis, opening the door to examining its clinical use in preventing MSG-induced cerebellar motor incoordination.

## 1 Introduction

Monosodium glutamate (MSG) is one of several forms of glutamic acid naturally found in foods, in addition to the characteristic umami taste (Yamamoto and Inui-Yamamoto, 2023). Whatever, the dietary source of glutamic acid which enters the blood stream for intestinal absorption is structurally symmetrical (Geha et al., 2000). Glutamate, a key excitatory neurotransmitter in the cerebellum, activates its receptors to mediate both physiological and pathological processes. When ingested as MSG, it elevates extracellular glutamate levels, potentially causing excitotoxicity, metabolic dysregulation, and neurological symptoms (e.g., headaches) in susceptible individuals, thereby disrupting normal physiological balance (Mattson, 2008; Rondi-Reig et al., 2014; Gallo et al., 1982). The cerebellum plays a crucial role in sensory perception integration and motor input, which controls and coordinates voluntary movement (Standring et al., 2005). Previous research has documented that oral administration of MSG at doses ranging from 3 to 6 g/kg per day for 14 days elevates extracellular glutamate to neurotoxic levels, causing overstimulation of NMDA/AMPA receptors that induces excitotoxic degeneration of Purkinje cells (Hashem et al., 2012; Eweka and OmIniabohs, 2007). MSG is believed to potentially cause a range of neurological symptoms in sensitive individuals, including the infamous Chinese restaurant syndrome. This condition is characterized by palpitations and numbness in the neck and back of the arms, highlighting the potential impact of MSG on our health (Kenney, 1986). Moreover, MSG induces severe neurotoxic effects like long-term depression via impaired synaptic plasticity and mGluR dysfunction (Calabresi et al., 1999), brain cell damage (Onaolapo et al., 2016), epilepsy through NMDA-mediated hyperexcitability and GABAergic inhibition loss (Stafstrom, 2004), and retinal degeneration via excitotoxic ganglion cell damage and Müller cell dysfunction (Babai et al., 2006). MSG triggers neurotoxicity via glutamate receptor overactivation (NMDA/AMPA), causing Ca^2+^ overload, mitochondrial dysfunction, and oxidative stress (ROS overproduction, lipid/DNA damage). This cascade activates apoptosis (via mitochondrial permeability transition pore (mPTP), caspases) (Farombi and Onyema, 2006).

Phosphatidylinositol 3-kinase (PI3K) is a heterodimer formed of two subunits: the catalytic subunit p110 with four isoforms (alpha, beta, gamma, and delta, known as PI3KCA, PI3KCB, PI3KCG, and PI3KCD) and the regulatory subunit p85, which is determined by three different genes (alpha, beta, and gamma) (Burke, 2018). Most cerebral cortex, cerebellar, and hippocampal neurons express the catalytic p110α and all the regulatory p85 subunits (Trejo and Pons, 2001; Chan and Ye, 2010). PI3K activates AKT, essential for synapse formation and neuronal development (Long et al., 2021). The protein kinase B (AKT) signaling pathway leads to neuronal cell survival by inhibiting neuronal apoptosis (through suppression of pro-apoptotic factors like BAD and caspase-9) and improving neurogenesis (via mTOR-dependent synaptic plasticity and CREB-mediated gene expression), as demonstrated in Parkinson’s and Alzheimer’s diseases (Long et al., 2021). Activation of PI3K/AKT can be regarded as the binding of neurotrophic factors like BDNF to its receptor TrKB (Gendy et al., 2023). , which subsequently promotes neuronal growth, differentiation, and survival.

Silymarin (SLM) is considered one of the extracts of Silybum marianum (Milk thistle) fruit; it is formed of one flavonoid and seven flavolignans that account for 65%–80% of milk thistle extract (Bijak, 2017). The literature has discussed its beneficial properties, including antioxidant, anti-inflammatory, antiproliferative, and anti-diabetic activities (Abdel-Moneim et al., 2015; Hosseinabadi et al., 2019; Stolf et al., 2017). Recent research has documented that SLM prevents the onset and progression of neurodegenerative disease via antioxidant (flavonoid-mediated ROS scavenging), anti-inflammatory mechanisms (Ashique et al., 2024; Hirayama et al., 2016). It was detected that encapsulating SLM in liposomes improves its bioavailability and solubility, protects it from gastric degradation, and enhances intestinal absorption via lipid bilayer fusion with epithelial cells (Kumar et al., 2014). Liposomes are formed of a closed lipid bilayer with an internal aqueous component. They can deliver small hydrophilic and lipophilic agents, nucleic acids, and large proteins, increasing drug therapeutic activity and safety (Nsairat et al., 2022; Abu Lila and Ishida, 2017). Elmowafy et al. (2013) reported that silymarin-loaded nanoliposomes provide better cellular absorption for SLM than free silymarin. To our knowledge scanty if any researches explore the effect of silymarin-loaded nanoliposomes on MSG-induced cerebellar toxicity, using mutli-panel investigations. This research aimed to explore the neurotoxic effect of MSG on rats’ cerebellum and its possible PI3K/AKT mechanistic pathway as well as measuring the protective effects of silymarin-loaded nanoliposomes.

## 2 Materials and methods

### 2.1 Experimental animals

Forty male Wistar rats weighing 180–220 g were grouped 2 weeks before the experiments for acclimatization. They were housed in a controlled experimental environment with humidity maintained at 60% ± 10%, temperature at 25°C ± 2°C, and a 12-h light/12-h dark cycle. Food and potable water were accessible *ad libitum*. This study was approved by the Research Ethics Committee of Umm Al-Qura University, Makka, Saudi Arabia, with code number HAPO-02-K-012-2024-01-1949, under the animal research reporting of *in vivo* experiments (ARRIVE) guidelines.

### 2.2 Silymarin-nanoliposome (SLNPs) preparation

To prepare a 10 mL solution of SLNPs, the researchers mixed 8 mL of ethanol with 0.4 mL of 0.5 mM cholesterol and 1.6 mL of 0.5 mM DPPC. Ethanol was used as the primary solvent to dissolve these components due to its ability to efficiently solubilize lipophilic molecules. Subsequently, 0.1 mL of 1 mM SLM solution was added, ensuring uniform dispersion. The solution was bathed and sonicated (at 80 MHz) for 40 min to facilitate nanoparticle formation. Then, 10 mL of water was added and heated in the water bath to 60°C, allowing gradual removal of ethanol and stabilization of liposomal structures. The new mixture solution was mixed with deionized water, and the probe was sonicated for 40 minutes again to enhance nanoparticle uniformity. Finally, rotary evaporation (above the phase transition temperature, i.e., 50°C) was performed to completely remove residual ethanol, ensuring the final nanoparticle dispersion was free from organic solvent contamination. Blank liposome nanoparticles (BLNPs) were similarly created when no SLM solution was loaded, following the same procedure (Farooq et al., 2022).

### 2.3 Silymarin-nanoliposome characterization

The inner morphology of SLNPs was screened using the electron microscope (TEM, JEOL JEM-2100, Tokyo, Japan) operating at 200 kV. Images were analyzed using Digi-tal Micrograph and Soft Imaging Viewer software (Gatan Microscopy Suite Software, version 2.11.1404.0). The Z average (mean vesicular size), the polydispersity index (PDI), and the Z-potential (external charge) of SLNPs were measured using a Zetasizer Nano ZS analyzer.

### 2.4 Study design

The rats in the current study were divided into four experimental groups (n = 10): Group I consisted of control rats receiving 2 mL of 0.9% NaCl solution intraperitoneally; Group II comprised SLNPs rats receiving 500 μg/kg bw of SLNPs orally for 10 days by gavage. The selected dose was based on the previous study conducted by Maryam et al. (2023); and Group III consisted of MSG rats receiving an intraperitoneal injection of 3.5 mg/kg bw of MSG dissolved in 2 mL of 0.9% NaCl solution daily for 10 days to avoid crystallization. The selected dose was based on the previous work by Prast et al. (2015). MSG was obtained from El Nasr Pharm. Chem. Co., Egypt, available in powder form with a concentration of 99%; Group IV comprised rats receiving both MSG and SLNPs treatments according to the previously mentioned doses and route of administration. The therapeutic dose of SLNPs was chosen based on a previous reference study and a pre-experimental pilot study on multiple doses (100 μg/kg bw, 300 μg/kg bw, and 500 μg/kg bw) conducted to determine the effective dose, which revealed that 500 μg/kg provided maximum therapeutic benefits on cerebellar oxidative stress markers, and inflammatory cytokines level with minimal toxicity.

### 2.5 Motor coordination test

A rotarod apparatus (Ugo Basile model 7,700, Italy) designed for rats was used to examine their motor coordination. The rotarod test was determined according to the method of Prast et al. (2015). The test began by putting the rats on the rotarod for 1 minute to familiarize them with the apparatus. After that, the rats were removed. Subsequently, the rotarod was turned at a speed of 16 rotations per minute, and rats were put on it in the opposite direction of the rotation. Rats started to walk to avoid falling from the rotarod. Two parameters were recorded: the number of falls and the latency of rats on the running surface. The test was performed on three different days: day 1 (1 day before the treatment), day 12 (1 day after the treatment), and day 32 (21 days after the treatment conclusion). Each test was performed three times a day, lasting 180 s and with an interval of 1 hour between sessions. The number of falls every day was recorded as the average across the three sessions. The latency data were calculated by dividing the most extended two times spent on a rotarod by 180 s and presenting it as a percentage (Candelario-Jalil et al., 2004). The investigator performing the neuro-logical evaluation and rotarod test did not know the identity of the experimental groups until the data analysis was completed.

### 2.6 Preparation of cerebellar tissue and histological examination

At the end of motor coordination tests, blood samples were collected from the retro-orbital vein under anesthesia prior to decapitation. The blood was allowed to clot at room temperature and then centrifuged at 3,000 rpm for 10 minutes to separate the serum. The serum was aliquoted and stored at −80°C for subsequent assessment of systemic proinflammatory markers using enzyme-linked immunosorbent assay (ELISA). Following serum collection, rats were sacrificed by decapitation using ketamine (150 mg/kg bw). Subsequently, their brains were carefully removed, and the cerebellum was dissected. The cerebellum was divided into three portions: one portion was homogenized using a tissue homogenizer in an ice-cold phosphate buffer (pH 7.4) to maintain enzyme stability. The homogenization was carried out under cold conditions to prevent degradation of sensitive biochemical components. Following homogenization, the samples were centrifuged at 12,000 rpm for 10 min at 4°C, and the supernatant was collected to assess oxidative stress markers. The second portion was stored in 10% buffered neutral formalin for 24 h and then immersed in a paraffin block. Subsequently, 5 μm thickness sections were collected on a glass slide and deparaffinized for histological and immunohistochemical (IHC) examination. The third portion was stored at −80°C for biochemical and molecular examination.

### 2.7 Oxidative marker assay

Cerebellar tissue homogenate was assessed for levels of total antioxidant capacity (TAC) (catalog number: TA 2513), catalase (CAT) (catalog number: CA2517), glutathione (GSH) (catalog number: GR 2511), and superoxide dismutase (SOD) (catalog number: SD2521) levels by using commercially available kits from Bio Diagnostic, Cairo, Egypt, according to the manufacturer’s instructions.

### 2.8 Immunohistochemical assessment

According to the manufacturer’s instructions, the avidin-biotin complex (ABC) technique was used for immunostaining of Nrf2, GFAP, NF-κB, and caspase-3. On charged slides, 5 µm section slides were deparaffinized. Subsequently, all sections were heated for 10–20 min in a microwave with 10 mM citrate buffer; after that, the slides were treated with 0.3 mM hydrogen peroxide for 30 min to quench the endogenous peroxidase activity. The slides were then incubated with the primary anti-bodies, including anti-Nrf2, anti-GFAP, anti-NF-κB p65, and anti-caspase-3 (catalog numbers: PA5-27882, 13-0300, PA5-27617, and 43-7,800). Finally, the DAB (Catalogue number: 34,002, Thermo Fisher Scientific, Waltham, MA, United States) immunostaining level was calculated in ImageJ by two blind pathologists. To validate the specificity of our immunostaining, a control experiment was conducted using only the secondary antibody, without the primary antibody, under identical conditions. This step ensured the absence of non-specific binding or secondary antibody signals. The results confirmed that the immune reaction occurs exclusively with the primary anti-body, thereby ensuring the reliability and specificity of the immunohistochemical data. High-resolution images of complete cerebellar tissue sections were analyzed using ImageJ software (FIJI, National Institutes of Health, United States, version 1.54k). The digital image was converted to an 8-bit grayscale format and adjusted to highlight the positive areas. Binary thresholding and watershed segmentation were then applied to the thresholded image. The actual positive cells within the selective positive area (ROI) were counted using the ‘Analyze Particles’ tool, ensuring the identification of positive cells while excluding unwanted objects. The software calculated key parameters such as integrated density and area fraction of stained pixels. The results were statistically analyzed and expressed as the number of actual positive cells per positive area, presented as mean ± SD (Helps et al., 2012).

### 2.9 Enzyme-linked immunosorbent assay (ELISA)

The parameters TNFα, IL-6, IL-1β, BDNF, p-PI3K, and p-AKT were measured in cerebellar homogenate, and serum levels of TNFα, IL-6, and IL-1β using commercial enzyme-linked immunosorbent assay (ELISA) kits. These measurements were conducted following the manufacturer’s guidelines (catalog numbers: MBS175904, MBS2701082, MBS2023030, ERBDNF, MBS260381, MBS775153, CSB-E11987r, CSB-E04640r, and E-ER0012, respectively). To preserve the phosphorylated state of p-PI3K and p-AKT, the cerebellar homogenates were prepared in lysis buffer containing phosphatase inhibitors, as recommended. The phosphatase inhibitor cocktail used included sodium orthovanadate and okadaic acid to ensure accurate measurements of phosphorylated proteins.

### 2.10 Quantitative real-time PCR (qRT-PCR)

According to the manufacturer’s guidelines, total cerebellar RNA was extracted using the RNeasy Kits and QIAwave RNA Kits (Qiagen, Hilden, Germany). The RNA content was assessed using Thermo Fisher Scientific’s Nanodrop 8,000. Table 1 displays the list of primers used in the present study. The reaction was conducted in a 25 µL running volume comprising 10 µL of the 2× HERA SYBR® Green RT-qPCR Master Mix (Willow Fort, Nottinghamshire, United Kingdom), 0.5 µL of each primer at a concentration of 20 pmol, 1 µL of the RT Enzyme Mix (20×), 3 µL of RNA template, and 5 µL of RNAse-free water. Subsequently, experiments were conducted using a Step-One real-time PCR instrument. Reverse transcription occurred at 50°C for 30 min. For amplification, cDNA was denatured at 94°C for 15 min, followed by 40 cycles of 95°C for 15 s and 60°C for 30 s. Gene expression variation in the RNA samples was evaluated using the following method: the Ct of each sample was calculated using the 2^−ΔΔCT^ method and normalized to those of β-actin as the housekeeping gene.

**TABLE 1:** List of primer sequences used for RT-qPCR analysis.

Gene	Forward primer (5′→3′)	Reverse primer (5′→3′)	Accession NO.	Product size (bp)	Tm (°C)
HO-1	AGAGTTTCTTCGCCAGAGGC	ATCAAACAGAGGTCGCATGC	NM_012580.2	505	60
Bax	GTTGCCCTCTTCTACTTTG	AGCCACCCTGGTCTTG	NM_016993.2	505	60
Bcl-2	GTACCTGAACCGGCATCTG	ATCAAACAGAGGTCGCATGC	NM_016993.2	97	58.5
TrKB	GCTGACGAGTTTGTCCAGGA	TGGCTCCGTTGTAGAACCAC	NM_012731.3	590	60
β-actin	TCAACACCCCAGCCATGTAC	AATGCCTGGGTACATGGTGG	NM_031144.3	548	60

### 2.11 Statistical analysis

The current investigation data were analyzed using GraphPad Prism (Version 8.0, San Diego, CA, United States) and expressed as mean ± standard deviation (M ± SD). Significance comparisons between different experimental groups were assessed using a one-way analysis of variance (ANOVA), followed by Tukey’s *post hoc* test. A p-value less than 0.05 was considered significant.

## 3 Results

### 3.1 SLNPs characterization

**FIGURE 1:**
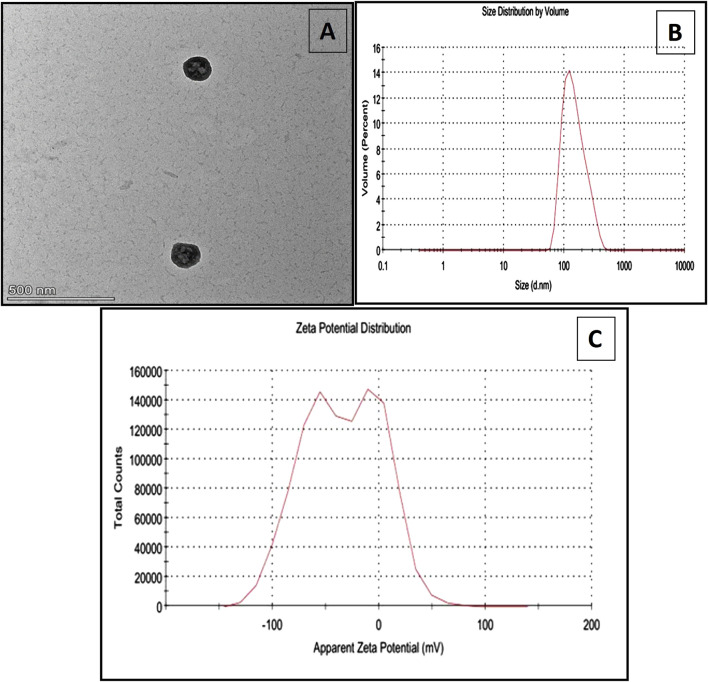
**(A)** the TEM characterization of SLNPs using a JEOL-JEM 2100 microscope, **(B)** the size distribution of the SLNPs, and **(C)** the zeta potential distribution of silymarin-loaded nanoliposomes.

TEM characterization of SLNPs using a JEOL-JEM 2100 microscope revealed their spherical morphology (Figure 1A). The average Zeta size distribution of SLNPs showed a mean diameter of 158.9 nm with a PDI of 0.165 (Figure 1B). The Zeta potential dispersion had a mean value of −34 mV (Figure 1C).

### 3.2 Positive impact of SLNPs on MSG-induced motor incoordination

Administration of MSG at a dose of 3.5 g/kg bw/day significantly increased the number of falls in the motor rotor test on days 1, 12, and 32 (F (3, 20) = 368.9, F (3, 20) = 446.8, F (3, 20) = 119.9, p < 0.05) and simultaneously decreased the two latency values in the rotarod test on days 1, 12, and 32 (F (3, 20) = 71.32, F (3, 20) = 226.5, F (3, 20) = 51.95, p < 0.05), respectively, compared to the control group, as shown in Tables 2, 3. Meanwhile, MSG rats that received SLNPs showed significantly improved motor function (p < 0.05), as evidenced by decreased motor fall times and increased latency values compared to MSG-only intake. This finding demonstrates the beneficial effect of SLNPs on MSG-induced motor incoordination.

**TABLE 2:** Total number of falls by rats during the rotarod test.

Days	Control	SLNPs	MSG	MSG + SLNPs
Day 1	0.48 ± 0.04	0.51 ± 0.05	3.77 ± 0.18^^^^###^	2.13 ± 0.34^^^^###$$$^
Day 12	0.82 ± 0.12	0.87 ± 0.13	6.29 ± 0.30^^^^###^	3.73 ± 0.48^^^^###$$$^
Day 32	3.42 ± 0.34	3.79 ± 0.40	11.76 ± 1.32^^^^###^	7.79 ± 1.11^^^^###$$$^

All data are expressed as M±SD. The notation ^^^p < 0.05 denotes significance versus the control group, ###p < 0.05 denotes significance versus the SLNPs group, whereas $$$p < 0.05 indicates relevance versus the MSG group.

**TABLE 3:** Two best latency values (as a percentage of 180 s) spent on the rotarod of the rats.

Days	Control	SLNPs	MSG	MSG + SLNPs
Day 1	83.40 ± 5.37	85.73 ± 5.31	44.10 ± 3.47^^^^###^	65.2 ± 7.50^^^^###$$$^
Day 12	72.88 ± 2.92	74.75 ± 3.29	29.05 ± 2.35^^^^###^	48.83 ± 5^^^^###$$$^
Day 32	53.43 ± 5.80	55.95 ± 2.51	22.84 ± 2.51^^^^###^	40.38 ± 5.68^^##$$$

All data are expressed as M ± SD. The notation ^^^p, ^^p < 0.05 denotes significance compared to the control group, ###p < 0.05 denotes significance compared to the SLNPs group, whereas $$$P < 0.05 indicates significance compared to the MSG group.

### 3.3 Impact of SLNPs on cerebellar histology

**FIGURE 2:**
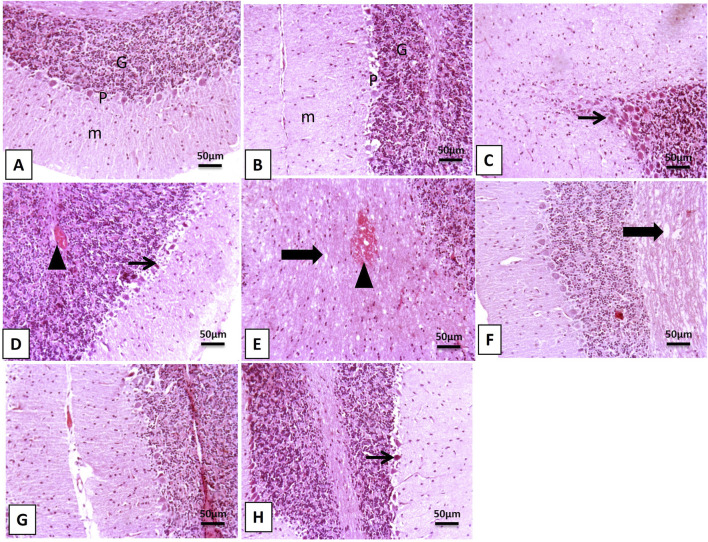
Photomicrographs of cerebellar sections. **(A,B)** The control and SLNPs groups exhibit normal histology of the cerebellar layers, including the molecular (M), Purkinje (P), and granular **(G)** cell layers. **(C)** In the MSG group, Purkinje cells appear shrunken and angulated with cytoplasmic eosinophilia and karyopyknosis (thin arrow), surrounded by a mild number of glial cells. **(D)** The MSG group shows mild spongiosis in the molecular layer, a shrunken necrotic Purkinje cell layer (thin arrow) surrounded by proliferated glial cells, and a thickened granular layer with congested blood vessels (arrowhead). **(E)** In the MSG group, a focal area of hemorrhage is observed as an arrowhead in the vacuolated white matter layer (thick arrow). **(F–H)** The MSG + SLNPs group shows normal architecture in up to 90% of cerebellar layers, except for the white matter layer, which exhibits mild to moderate spongiosis (thick arrow) and occasional shrunken Purkinje cells (thin arrow). Image magnification: 100×; inset = 400×.

The H&E examination of cerebellar sections from the control and SLNPs groups revealed a normal histological appearance, including intact molecular, Purkinje, and granular cell layers (Figures 2A,B). Meanwhile, oral MSG intake significantly distorted cerebellar histology, showing Purkinje cell shrinkage, cytoplasmic karyopyknotic eosinophilia, mild spongiosis in the molecular layer surrounded by proliferated glial cells, and thickening of the granular layer with congested blood vessels (Figures 2C–E). In contrast, SLNPs improved the histological structure of all cerebellar layers, with focal areas of hemorrhage observed in vacuolated white matter (Figures 2F–H).

### 3.4 Antioxidant effect of SLNPs against MSG-induced cerebellar oxidative stress

**FIGURE 3:**
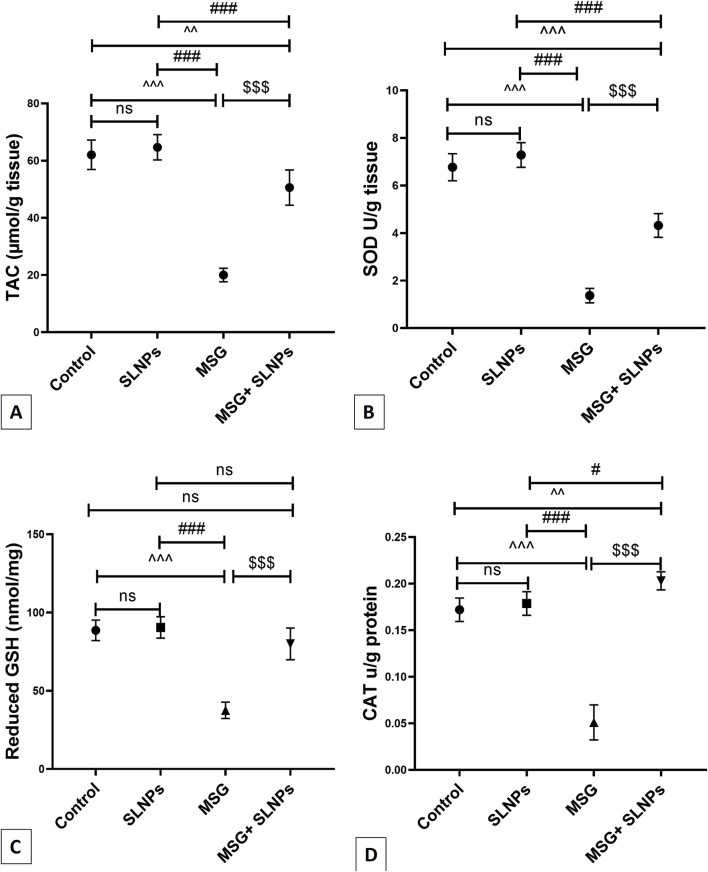
The effect of SLNPs and MSG on the cerebellar levels of oxidative stress markers. **(A)** TAC, **(B)** SOD, **(C)** GSH, and **(D)** CAT. The data are expressed as mean ± standard deviation (M ± SD), and statistical significance was determined using one-way ANOVA followed by Tukey’s *post hoc* test. Statistical significance is denoted as ^^^p, ^^p < 0.05 versus the control group, ^###^p, #p < 0.05 denotes significance compared to the SLNPs group, and $$$p < 0.05 versus the MSG group.

**FIGURE 4:**
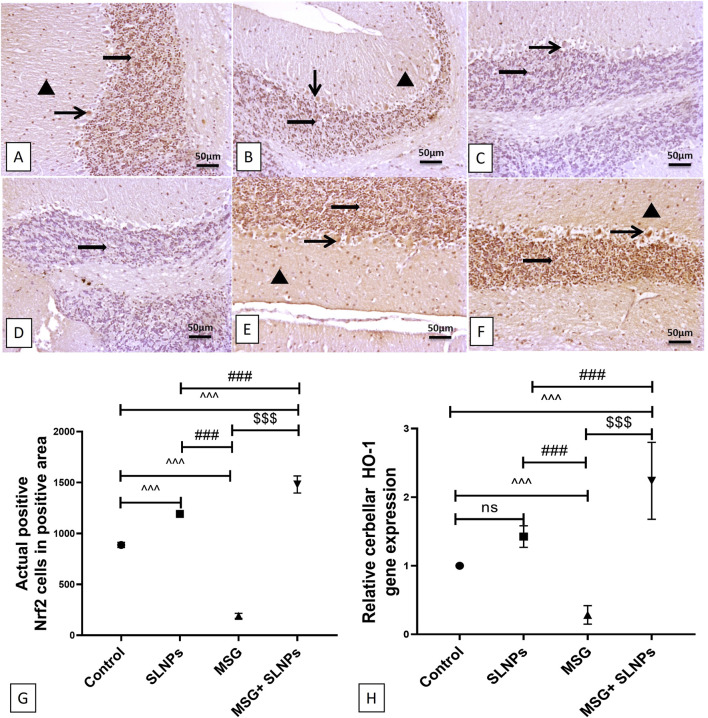
Representative IHC expression of Nrf2. **(A,B)** The control and SLNP groups (n = 10) exhibiting high expression of Nrf2 in the granular layer and Purkinje cell layer, with moderate expression in molecular neuronal cells. **(C,D)** The MSG group (n = 10) displaying faint expressions in the granular cell layer and Purkinje cell. **(E,F)** MSG + SLNPs (n = 10) demonstrating marked, intense immunopositive staining in molecular, Purkinje, and granular cell layers. Image magnification is 100x. The arrowhead indicates positive immunostained cells in the molecular layer; the thin arrow denotes positive Purkinje cells, and the thick arrow represents positive granular cells. **(G)** Histogram of actual positive Nrf2 cells in positive area. **(H)** Gene expression of HO-1 in cerebellar samples from different groups. All data are expressed as M ± SD. The data were analyzed for statistical significance using one-way ANOVA, followed by Tukey’s *post hoc* test for pairwise comparisons. Statistical significance is indicated as ^^^p < 0.05 vs. the control group; ^###^p < 0.05 vs. the SLNPs group, and $$$p < 0.05 vs. the MSG group.

As depicted in Figure 3, antioxidant markers TAC, SOD, GSH, and CAT significantly decreased in MSG-treated rats (F (3, 20) = 112.3, F (3, 20) = 189.2, F (3, 20) = 67.45, F (3, 20) = 145.2, p < 0.05), with reductions of 20 ± 2.36 (32.2%), 1.36 ± 0.30 (20.11%), 37.55 ± 5.24 (42.3%), and 0.05 ± 0.01 (29.4%), respectively, compared to the control rats. A significant (p < 0.05) recovery was observed in group IV rats that received SLNPs along with MSG, as evidenced by elevated levels of TAC and SOD, as well as increased GSH and CAT activity by 50.75 ± 6.17 (252.8%), 4.31 ± 0.49 (316.9%), 79.97 ± 10.11 (212.9%), and 0.20 ± 0.00 (400%), respectively, compared to the MSG group (Figures 3A–D). Furthermore, the MSG + SLNPs group exhibited a significant (p < 0.05) upregulation in antioxidant-regulating genes, as indicated by a marked increase in the immunoexpression of Nrf2 and gene expression of HO-1 by 1,481 ± 83.74 (780%) and 2.23 ± 0.56 (796.4%), respectively (Figures 4E–H), in contrast to the MSG group, which exhibited levels of 190.5 ± 25.27 (21.4%) and 0.28 ± 0.13 (28%) (Figures 4C,D,H), compared to the control groups (Figures 4A,B,H). Interestingly, SLNPs provided strong antioxidant protection against MSG-induced cerebellar oxidative insults.

### 3.5 SLNPs mitigate MSG-induced cerebellar inflammation

**FIGURE 5:**
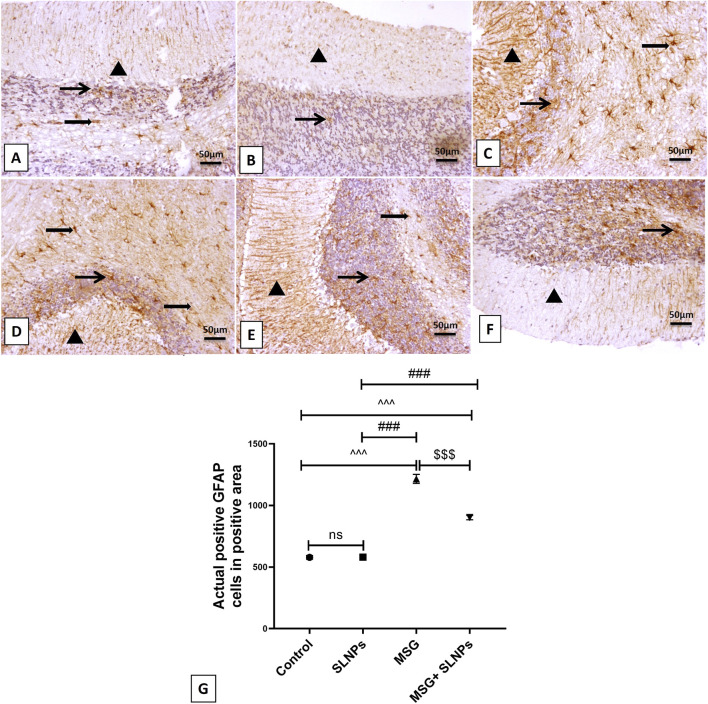
The IHC expression of GFAP. **(A,B)** represent the control and SLNP groups (n = 10), displaying a mild expression of GFAP in the molecular, granular, and white matter cell layers. **(C,D)** depict the MSG group (n = 10) with marked, intense astrocytic immunoreactivity across the molecular, granular, and white matter cells. **(E,F)** show the MSG + SLNPs group (n = 10) with moderate immunopositive astrocytic staining in the granular cell layer and white matter layer, along with more fiber staining in the molecular cell layer. Arrowheads denote positive immunostained areas in the molecular layer; thin arrows indicate positive immunostained cells in the granular cell layer; and thick arrows point to positive astrocytes in the white matter cell layer. **(G)** Histogram of actual positive GFAP cells in positive area. All data are expressed as M ± SD. Differences were evaluated for statistical significance through one-way ANOVA, with Tukey’s test applied for *post hoc* analysis. Image magnification is set at 100x. ^^^p < 0.05 indicates statistical significance versus the control group, ^###^p < 0.05 signifies significance versus the SLNPs group, while $$$p < 0.05 signifies significance versus the MSG group.

**FIGURE 6:**
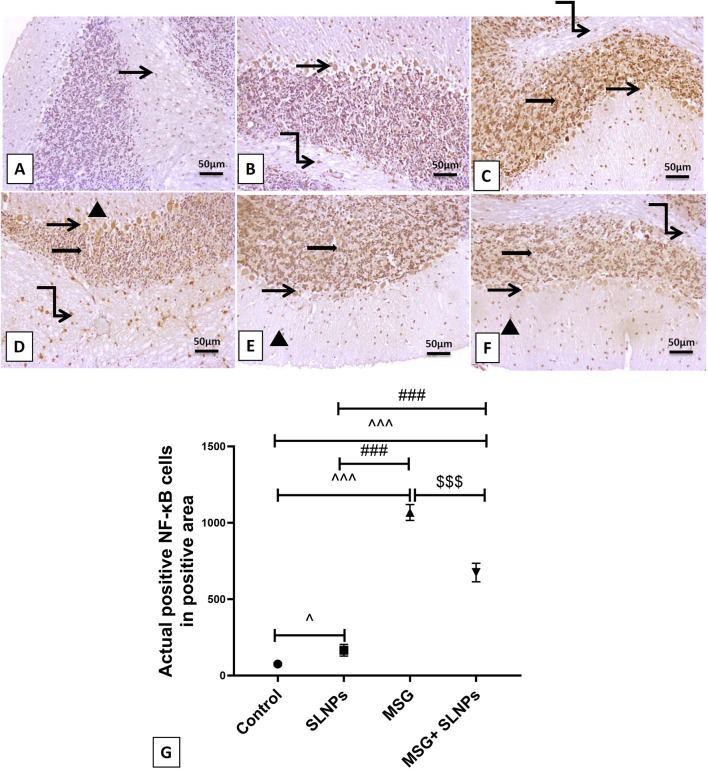
The IHC expression of NF-κB across different treatment groups. In the control group (n = 10) **(A)**, minimal NF-κB expression is observed in white matter cells without ex-pression in the granular and Purkinje cell layer or molecular neuronal cells. In the SLNPs group (n = 10) **(B)**, mildly immunopositive stained cells are noticed in the Purkinje (more cytoplasmic) and granular and white matter cells. The MSG group (n = 10) **(C,D)** exhibits marked, intense expression in the granular cell layer, Purkinje cells with moderate to high expression in the molecular cell layer, and white matter cells. In the MSG + SLNPs group (n = 10) **(E,F)**, moderately faintly immunopositive staining is observed in Purkinje (more cytoplasmic) and granular cell layers, with less expression in molecular and white matter layers. Magnification for images is 100x. Notations include arrowheads for positive immunostained cells in the molecular layer, thin arrows for positive Purkinje cells, thick arrows for positive granular cells, and twisted arrows indicating immunopositive stained white matter cells. **(G)** Histogram of actual positive NF-κB cells in positive area. Data are expressed as M ± SD. One-way ANOVA was performed to determine significance, supplemented by Tukey’s *post hoc* test for detailed group comparisons. Statistical significance is represented as ^^^p, ^p < 0.05 compared to the control group, ^###^p < 0.05 versus the SLNPs group, and $$$p < 0.05 versus the MSG group.

**FIGURE 7:**
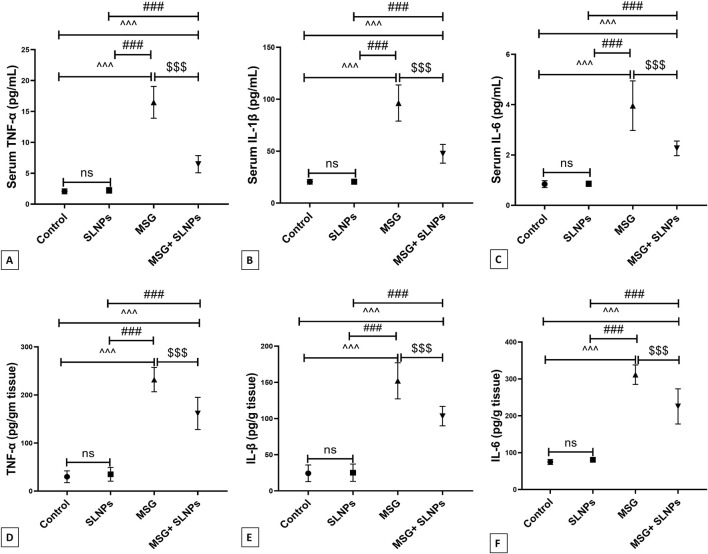
Serum **(A–C)** and cerebellar protein levels **(D–F)** of proinflammatory markers TNF-α, IL-1β, and IL-6 using ELISA across different groups. Data are presented as M ± SD. One-way ANOVA with Tukey’s *post hoc* test was used to evaluate statistical significance between groups. Statistical significance is represented as ^^^p < 0.05 versus the control group, ^###^p < 0.05 versus the SLNPs group, and $$$p < 0.05 compared to the MSG group.

MSG intraperitoneal injection intake induced severe cerebellar inflammation by a significant increase in the immunoexpression of GFAP, NF-κB (F (3, 20) = 1,031, F (3, 20) = 650.8, p < 0.05), and serum and cerebellar protein levels of proinflammatory markers TNF-α, IL-1β, and IL-6 (F (3, 20) = 123.0, F (3, 20) = 77.74, F (3, 20) = 48.28, F (3, 20) = 111.6, F (3, 20) = 87.71, F (3, 20) = 105.4, p < 0.05) by 1,215 ± 36.53 (2,100.2%), 1,067 ± 52.00 (1,425.8%), 3.96 ± 0.98 (471.4%), 14.99 ± 0.04 (632.4%), 19.93 ± 0.54 (473.3%), 232 ± 25.31 (773.3%), 152.2 ± 24.96 (625.5%), and 311.5 ± 26.28 (416.2%), respectively (Figures 5C,D,G; Figures 6C,D,G; Figures 7A–F) in comparison to control rats (Figures 5A,B; Figures 6A,B; Figures 7A–F). Meanwhile, coadministration of SLNPs with MSG significantly (p < 0.05) decreased GFAP, NF-κB and cerebellar inflammatory cytokines by 901.3 ± 17.91 (74,2%), 674.2 ± 60.40 (63.2%), 6.46 ± 1.38 (39.1), 47.50 ± 9.02 (49.2%), 2.26 ± 0.29 (57%), 161.7 ± 33.48 (69.6%), 103.3 ± 13.40 (67.8%), and 225.3 ± 47.66 (72.3%) (Figures 5E,F; Figures 6E,F; Figures 7A–F). SLNPs exhibited good anti-inflammatory properties against MSG-induced inflammation.

### 3.6 SLNPs attenuate MSG-induced cerebellar apoptosis

**FIGURE 8:**
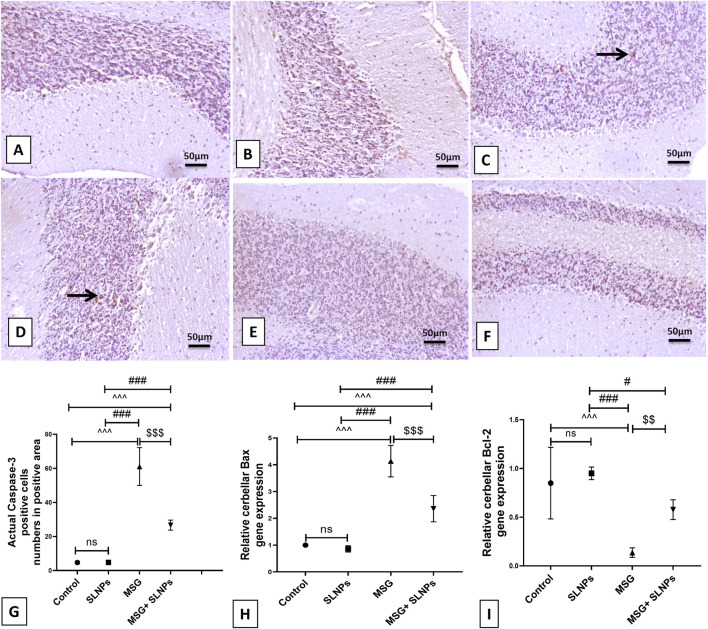
The IHC expression of caspase-3. **(A,B)** display negative immunopositive stained cells for control and SLNPs (n = 10). **(C,D)** depict moderate immunopositive staining in the granular cell layer for the MSG group (n = 10). **(E,F)** demonstrate negative immuno-positive staining cells in all cerebellum layers for MSG + SLNPs (n = 10). H and I illustrate the gene expression of Bax and Bcl-2. Image magnification is 100x, with the inset at 400x. Thin arrows indicate immunopositive stained cells. **(G)** Histogram of actual positive caspase-3 cells in positive area. **(H,I)** Gene expression of Bax and Bcl-2 in cerebellar samples from different groups. All data are introduced as M ± SD. Statistical analyses were conducted using one-way ANOVA, with subsequent Tukey’s *post hoc* testing to determine intergroup significance. Statistical significance is denoted as ^^^p < 0.05 in comparison to the control group ^###^p, #p < 0.05 in comparison to the SLNPs group, and $$p < 0.05 in comparison to the MSG group.

Caspase-3 immunoexpression significantly increased (F (3, 20) = 127.8, p < 0.05) in MSG groups by 61.10 ± 11.09 (1,297%) (Figures 8C,D,G) compared to control rats (Figures 8A,B). MSG elevated the gene expression of Bax and decreased Bcl-2 (F (3, 20) = 93.12, F (3, 20) = 20.9, p < 0.05) by 4.13 ± 0.58 (413%) and 0.13 ± 0,04 (152.9%), respectively (Figures 8H,I). Furthermore, the combined intake of MSG and SLNPs significantly (p < 0.05) reduced the level of positive immunostained apoptotic cells and the gene expression of Bax by 26.67 ± 2.94 (43.6%) and 2.36 ± 0,48 (57.1%), respectively, while increasing the gene expression of Bcl-2 by 0.58 ± 0.10 (446.1%) compared to the MSG group (Figures 8E–I). SLNPs exhibited potent anti-apoptotic effects against MSG-induced cerebellar apoptosis.

### 3.7 SLNPs reverse MSG-induced alternation in BDNF, TrKB, p-PI3K, and p-AKT

**FIGURE 9:**
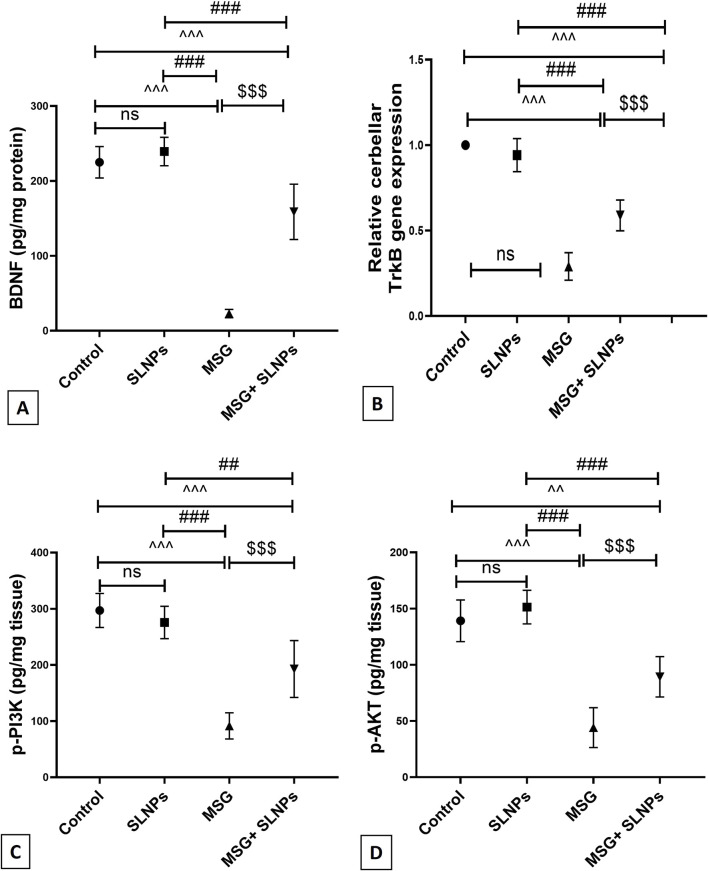
**(A)** The cerebellar protein level of BDNF by ELISA across different groups, **(B)** the gene expression of TrkB, and **(C,D)** the protein levels of p-PI3K and p-AKT. All data are represented as M ± SD. Statistical significance was evaluated by one-way ANOVA, followed by Tukey’s *post hoc* test for multiple comparisons. Statistical significance is denoted as ^^^p, ^^p < 0.05 versus the control group, ^###^p, ##p < 0.05 compared to the SLNPs group, and $$$p < 0.05 versus the MSG group.

The protein level of BDNF and gene expression of its receptor TrkB significantly decreased by 23 ± 5.5 (102,2%) and 0.29 ± 0.08 (290%), respectively (Figures 9A,B), with a statistical significance of p < 0.05. Furthermore, the protein levels of p-PI3K and p-AKT, determined by the ELISA assay significantly decreased by 91.67 ± 23.28 (30.8%) and 44.17 ± 17.72 (31.7%) (Figures 9C,D), respectively, compared to control groups, similarly at p < 0.05. On the other hand, SLNPs significantly reversed the levels of BDNF, TrkB, p-PI3K, and p-AKT by 158.8 ± 36.88 (690.4%), 0.58 ± 0.09 (200%), 198.8 ± 50.57 (210.3%), 89.33 ± 17.93 (202.2%), respectively, concerning the MSG group, with statistical significance at p < 0.05.

## 4 Discussion

The study was designed to investigate the neuroprotective effect of SLNPs against MSG neurotoxicity by targeting the PI3K/AKT axis. The following findings support this notion: 1) improvement of motor coordination in treated rats; 2) preservation of the histological structure of the cerebellum; 3) activation of BDNF and its receptor TrkB, triggering the PI3K/AKT axis; 4) activation of the PI3K/AKT axis; 5) enhancement of the endogenous antioxidant system (TAC, SOD, GSH, CAT) due to upregulation of the Nrf2/HO-1 antioxidant gene inducer; 6) inhibition of the nuclear NF-κB inflammatory transcription factor, with a reduction in the supernatant levels of TNF-α, IL-6, IL-1β inflammatory cytokines, and a marked decrease in GFAP active astrocytes; and 7) a decrease in apoptotic markers caspase-3 and Bax, with an increase in the Bcl-2 anti-apoptotic marker.

The hydrophilic character of SLM encourages its incorporation into bilayer liposomal vesicles, thereby increasing its bioavailability and circulation time (Immordino et al., 2006). Liposomal encapsulation improves the solubility and stability of SLM, allowing for better absorption and prolonged systemic exposure. Studies have shown that liposomal formulations increase the dissolution rate and protect SLM from rapid degradation, leading to higher plasma concentrations and extended therapeutic effects (Yang et al., 2015). Additionally, lipid-based delivery systems, such as liposomes, facilitate targeted delivery, reducing the required dosage while maintaining efficacy (Ranjbar et al., 2023). The standard size of unloaded liposomes is 24 nm, while the silymarin-loaded liposome size was measured at 158.9 nm, indicating successful drug loading (Arif et al., 2022; Farooq et al., 2022). However, the zeta potential measurement of SLNPs is −34 mV. Zeta potential measurements provide valuable insights into the extent of medication loading on nanoparticles, essential for maintaining stability and preventing aggregation. For optimal nanoparticle stability, a zeta potential of −30 mV or higher is considered ideal, as it enhances electrostatic repulsion between particles, reducing the likelihood of aggregation (Seibert et al., 2019). Thus, the increase in the zeta potential charge of SLNPs to −34 mV indicates improved liposomal stability and dispersion, ensuring effective drug delivery.

Intraperitoneal injection of 3.5 mg/kg bw of MSG disrupted the histological structure of the cerebellum, resulting in marked shrinkage of the Purkinje cells, evident pyknosis, gliosis in the molecular layer, and a thickened, congested granular cell layer. The damage to the Purkinje cell layer led to motor incoordination, as evidenced by increased rat falls and a decrease in the mean latency percentage spent on the rotarod. This finding aligns with previous work by Prast et al. (2015), demonstrating that 3.5 mg/kg bw of MSG was sufficient to induce Purkinje cell damage associated with motor incoordination. Similarly, Hashem et al. (2012) found that 3 mg/kg bw per day of MSG resulted in Purkinje cell damage.

The study by Eweka and OmIniabohs (2007) observed that administering 3–6 g of MSG mixed with rats’ food damaged the Purkinje and granular cell layers. In contrast, the study by Aminuddin et al. (2015) revealed that 2 mg/kg bw did not produce a significant difference in Purkinje cell count between the MSG and control rats. This can be regarded as a small dose of MSG. On the other hand, combined administration of SLNPs improved the morphological appearance of the Purkinje cell layer, decreasing pyknosis and enhancing motor coordination performance. This finding is consistent with the study by Badawy Khair and Mohammed (2021), which reported the ameliorative effects of SLM and Coen-zyme-Q10 on atherosclerosis-induced alterations in the cerebellar Purkinje cell layer. However, SLNPs exert neuroprotective effects by upregulating antioxidant pathways, such as Nrf2/HO-1, which mitigate oxidative stress and neuronal apoptosis. Additionally, SLNPs stimulate the BDNF/TrKB signaling axis, promoting neuronal survival and synaptic plasticity. This activation restores Purkinje cell density and dendritic complexity, thereby improving cerebellar function and motor coordination. Research suggests that SLNPs enhance cerebellar resilience, reducing MSG-induced damage and supporting long-term neuronal health.

The PI3K/Akt axis regulates cellular activities such as cell survival and apoptosis (Li et al., 2012), playing a crucial role in the defense against neurodegenerative diseases, including Parkinson’s disease (Levy et al., 2009; Goyal et al., 2023). AKT, as the downstream master protein of PI3K, contributes to cellular maintenance and proliferation (Long et al., 2021). Phosphorylation of the PI3K/AKT pathway is initiated by binding to certain neurotrophic factors (Rai et al., 2019). BDNF exerts a neuroprotective function after binding to its receptor TrKB, followed by subsequent phosphorylation (Jin, 2020). The BDNF/TrKB signaling axis activation promotes neurogenesis through AKT pathway stimulation (Bai et al., 2019). Upon BDNF binding to TrKB, a phosphorylation cascade is initiated, leading to the activation of PI3K, which subsequently stimulates AKT. This activation enhances neuronal survival, differentiation, and synaptic plasticity, contributing to neurogenesis and cognitive function maintenance. AKT also regulates transcription factors such as CREB, which promote the expression of genes involved in neuronal growth and repair. The BDNF/TrKB-mediated AKT pathway plays a vital role in protecting neurons from apoptosis and oxidative damage, reinforcing its significance in neuroprotection and brain function. The PI3K/Akt axis serves as a key regulator of neuronal survival, metabolic balance, and synaptic plasticity. Its activation enhances cellular resilience against oxidative stress and inflammation, both of which contribute to neurodegenerative conditions such as Parkinson’s disease and Alzheimer’s disease. Additionally, PI3K/Akt signaling controls critical downstream effectors, including GSK-3β, FOXO transcription factors, and NF-κB, which influence neuronal health and function. Dysregulation of this pathway has been implicated in neuronal apoptosis and synaptic dysfunction, making it a potential therapeutic target for neurodegenerative disorders. In the present investigation, it was found that MSG significantly decreased the protein levels of BDNF and the gene expression of its receptor TrKB, as well as markedly decreased the protein level of phosphorylated PI3K and its downstream AKT, compared to control rats, as determined by the ELISA assay. Consistent with the current finding, the study by Gürgen et al. (2021) reported that MSG significantly (p < 0.05) decreased the immunoexpression of BDNF in the hippocampal CA1 and DG regions. Additionally, Vucic et al. (2018) documented the effect of the PI3K/Akt inhibitor on MSG-induced thymocyte toxicity.

In contrast, SLNPs significantly increased the BDNF/TrKB signaling axis and PI3K/Akt pathway activation. These results align with a previous study by Song et al. (2016), which reported that SLM treatment improved learning and memory in lipopolysaccharide-treated rats through upregulation of the BDNF/TrkB pathway. Similarly, the study by Li et al. (2015) reported the positive effect of silibinin on the PI3K/Akt pathway in palmitate-induced insulin resistance in C2C12 myotubes.

The phosphorylated PI3K/Akt axis produces neuroprotection by activating down-stream prosurvival substrates such as Nrf2 (Liu et al., 2021). Moreover, it halts neuroinflammation and neuronal apoptosis by modulating downstream effectors, including NF-κB, FOX, GSK-3β (Cheong et al., 2020), and caspase-3 (Feng and Xi, 2022). The present results indicated that MSG decreases the immunoexpression of Nrf2 and the gene expression of HO-1 antioxidant response elements, which are essential for activating the endogenous antioxidant system and maintaining cellular redox homeostasis. Furthermore, MSG reduces the activity of SOD and CAT and the levels of GSH and TAC compared to the control group.

This finding aligns with a recent experiment by Hashem et al. (2012), which reported that intraperitoneal injection of MSG increases cerebellar GFAP immunoexpression. Similarly, Abu-Elfotuh et al. (2022) demonstrated the suppressive effect of MSG on hippocampal gene expression of Nrf2 and HO-1. Another study reported the negative im-pact of MSG on the hepatic Nrf2/HO-1 signaling pathway and the antioxidant activities of SOD, CAT, and GSH (Soliman et al., 2023). Fortunately, the coadministration of 500 μg/kg bw of SLNPs counteracted MSG-induced cerebellar toxicity by upregulating the Nrf2/HO-1 axis, which serves as a crucial antioxidant gene inducer by enhancing the transcription of key enzymes involved in oxidative stress defense. Specifically, Nrf2 activation triggers HO-1 expression, which mitigates oxidative damage by increasing cellular levels of GSH, TAC, SOD, and CAT. This molecular mechanism strengthens the endogenous antioxidant system, thereby preserving neuronal integrity and redox homeostasis. Additionally, the PI3K/Akt axis plays a vital role in facilitating Nrf2 activation, thereby enhancing antioxidant defenses. PI3K phosphorylation leads to Akt activation, which promotes the nuclear translocation of Nrf2, allowing it to bind to antioxidant response elements (AREs) and drive the expression of HO-1 and other protective enzymes. Furthermore, PI3K/Akt inhibits Keap1, a negative regulator of Nrf2, ensuring sustained activation of antioxidant responses. By modulating these molecular pathways, SLNPs contribute to the effective defense against MSG-induced oxidative damage, preventing neuronal apoptosis and cerebellar dysfunction. These results are consistent with previous studies that highlighted the beneficial effects of SLM on the Nrf2/HO-1 pathway in mitigating docetaxel-induced central and peripheral neurotoxicity, as well as the upregulatory effects of silymarin-encapsulated liposomes on SOD, CAT, and GSH levels in a rat model of diabetic nephropathy (Yardım et al., 2021; Chen et al., 2021). The antioxidant effect of SLNPs may be attributed to the flavonoid properties of SLM and its positive modulation of the PI3K/Akt axis, which activates the Nrf2/HO-1 pathway and stimulates the production of antioxidants.

The PI3K/Akt pathway exerts a prosurvival effect through its anti-inflammatory and anti-apoptotic regulation of NF-ĸB and caspase-3 (Cheong et al., 2020; Feng and Xi, 2022). Activation of PI3K leads to Akt phosphorylation, which enhances NF-ĸB signaling by facilitating the degradation of IĸB, an inhibitory protein that prevents NF-ĸB activation. This allows NF-ĸB to translocate into the nucleus, where it promotes the expression of anti-apoptotic genes, such as Bcl-2 and Bcl-xL, thereby ensuring neuronal survival and resilience against oxidative stress. In the present study, MSG significantly increases the immunoexpression of cerebellar NF-ĸB and GFAP while causing a pronounced increase in the serum and supernatant protein levels of TNF-α, IL-6, and IL-1β. Additionally, there is an increase in the immunoexpression of caspase-3 and the mRNA levels of Bax, accompanied by a decrease in Bcl-2. The upregulation of pro-inflammatory cytokines TNF-α, IL-6, and IL-1β is a hallmark of MSG-induced neuroinflammation, contributing to neuronal damage and synaptic dysfunction. MSG triggers excessive glutamate accumulation, leading to excitotoxicity, oxidative stress, and microglial activation, all of which amplify inflammatory responses. This inflammatory cascade results in neurotoxicity, disrupted cellular signaling, and increased neuronal apoptosis, worsening cerebellar dysfunction. These findings are in agreement with previous studies by Abu-Elfotuh et al. (2022), which reported that MSG significantly upregulates brain gene expression of NF-ĸB and the serum and protein levels of TNF-α, IL-1β, and GFAP. Furthermore, they observed an increase in brain Bax and a decrease in Bcl-2 gene expression.

Interestingly, oral administration of SLNPs with MSG demonstrated a potent anti-inflammatory effect, as evidenced by the significant downregulation of NF-κB, GFAP, TNF-α, IL-6, and IL-1β levels. Additionally, it exhibited an anti-apoptotic effect by markedly decreasing the apoptotic markers caspase-3 and Bax while enhancing the level of the anti-apoptotic marker Bcl-2. These findings are supported by previous studies conducted by Chen et al. (2021), which reported that SLNPs mitigate nephroinflammation in diabetic nephropathy rats by reducing both the protein level and gene expression of TNF-α and IL-6. Khan et al. (2014) documented that topical application of SLM suppresses dermal inflammation in chemical-induced skin carcinogenesis by downregulating the NF-κB transcription factor. Moreover,Gheybi et al. (2023) demonstrated the anti-apoptotic effect of SLNPs against cadmium-induced apoptosis in MRC-5 and A-549 cancer cells by decreasing caspase-3 expression.

Additionally, Wang et al. (2018) investigated the ameliorative effect of SLM against triptolide-induced hepatic apoptosis by reducing levels of cleaved caspase-3, cytochrome c, and Bax expression, as evidenced by immunoexpression, ELISA, and Western blotting. The antioxidative, anti-inflammatory, and anti-apoptotic properties of SLNPs are attributed to their inherent antioxidant capacity and their stimulatory effect on the PI3K/Akt axis, which leads to downregulation of the NF-κB transcription factor and the proapoptotic caspase-3. These combined effects explain the observed histological improvement in cerebellar tissue and the enhancement of motor coordination. One major limitation of our study is the lack of regional specificity in cerebellar sampling, particularly in relation to Purkinje cell morphology and molecular assessments. Given that different cerebellar regions have distinct functions and varying susceptibilities to excitotoxic damage, our interpretation of MSG-induced neurotoxicity and SLNP-mediated protection remains somewhat constrained. To address this, future studies will incorporate region-specific analyses, differentiating between vermis and hemispheric Purkinje cells to refine our understanding of cerebellar responses.

## 5 Conclusion

The study demonstrated the potent neuroprotective effects of SLNPs against MSG-induced cerebellar damage. SLNPs significantly mitigated oxidative stress by restoring antioxidant markers (TAC, SOD, GSH, and CAT) and upregulating antioxidant-regulating genes (Nrf2 and HO-1). Their anti-inflammatory properties were evident through reducing proinflammatory markers (GFAP, NF-κB, TNF-α, IL-1β, and IL-6) and improving cerebellar histology. Furthermore, SLNPs exhibited strong anti-apoptotic effects by modulating apoptotic markers (caspase-3, Bax, and Bcl-2) and effectively reversing disruptions in signaling pathways (BDNF, TrkB, p-PI3K, and p-AKT). Behavioral findings, such as enhanced motor coordination, further reinforce their therapeutic potential. Thus, the study concludes that SLNPs counteract MSG-induced cerebellar damage by targeting oxidative stress, inflammation, apoptosis, and disrupted signaling pathways, particularly emphasizing the PI3K/AKT pathway. These findings suggest that SLNPs represent a promising neuroprotective intervention against pollutant-induced neurodegeneration.

## Data Availability

The original contributions presented in the study are included in the article/supplementary material, further inquiries can be directed to the corresponding author.
